# Glyphosate Is Converted into Energy in a Microfluidic Fuel Cell Equipped with a Low-Content Ni Anode and a Metal-Free Cathode

**DOI:** 10.1021/acsomega.5c01606

**Published:** 2025-05-07

**Authors:** Willy Bellard Kira, Daniel F. Costa-Filho, Cinthia R. Zanata, Isabel M. C. de Alcantara, Jefferson Bettini, Flávio L. Souza, Heberton Wender, Cauê A. Martins

**Affiliations:** † Institute of Physics, 54534 Universidade Federal de Mato Grosso do Sul, CP 549, 79070-900 Campo Grande, MS, Brazil; ‡ Institute of Chemistry, Universidade Federal de Mato Grosso do Sul, CP 549, 79070-900 Campo Grande, MS, Brazil; § Brazilian Nanotechnology National Laboratory (LNNano), Brazilian Center for Research in Energy and Materials (CNPEM), 13083-100 Campinas, São Paulo, Brazil

## Abstract

Glyphosate, a widely used herbicide, poses significant environmental and health risks due to its persistence and potential toxicity. Existing mitigation methods often face challenges such as incomplete degradation or the generation of harmful byproducts, in addition to consuming energy to operate. Herein, we report the first demonstration of glyphosate being directly used as a fuel in a microfluidic fuel cell (μFC), enabling simultaneous energy generation and pollutant degradation. The μFC features a nickel-sputtered carbon paper (Ni/CP) anode and a metal-free carbon paper (CP) cathode. The sputtering process ensures the formation of well-dispersed, high-purity Ni nanoclusters, enhancing surface activity and catalytic performance with ultralow metal loading. Coupled with hypochlorous acid (HClO) reduction on the cathode, the μFC achieved a maximum power density of 0.18 mW cm^–2^ and glyphosate conversion efficiencies exceeding 99% for diluted solutions (16.2 ppm) and 82% for concentrated solutions (29.6 ppm). High-performance liquid chromatography confirmed the degradation of glyphosate to levels below the World Health Organization’s recommended limit of 0.9 mg L^–1^. Although additional research on the product of the μFC is necessary, this report on a membraneless μFC utilizing glyphosate as the sole energy source in a mixed-media environment shows energy recovery from an environmental pollutant under zero-bias conditions. This scalable, cost-effective system highlights the potential of integrating advanced nanostructured materials and electrochemical techniques for simultaneous pollutant removal and sustainable energy production.

## Graphical abstract

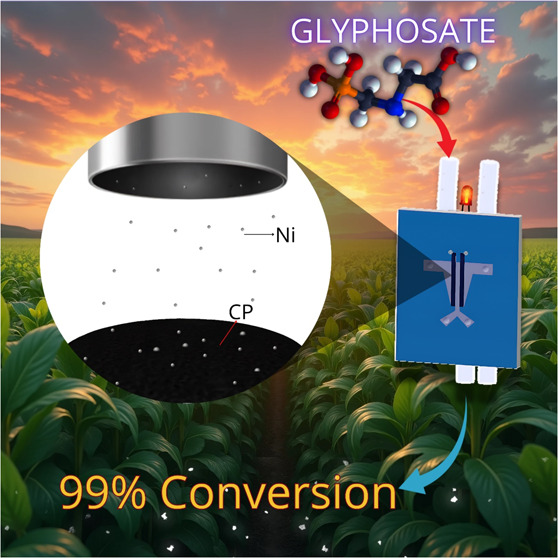

## 1 Introduction

Grass and broad-leaved weeds are commonly managed by using herbicides. However, the extensive use of these chemicals has resulted in significant environmental pollution, prompting growing concerns. Research indicates that less than 0.1% of herbicides used for pest control reach their intended targets.^1^ Consequently, over 99% of these chemicals are released into the environment, potentially contaminating soil, water, and atmospheric ecosystems.^1^ Glyphosate (C_3_H_8_NO_5_P), one of the most widely used herbicides globally, is projected to be applied in quantities of approximately 1.35 million tons annually.^2^ The World Health Organization has classified glyphosate as a Group 2A substance, indicating it is probably carcinogenic to humans.^3,4^

Several techniques have been proposed to mitigate glyphosate pollution, including physical, biological, and chemical processes.^5–7^ Physical methods primarily involve adsorption, where contaminants adhere to the surface of a solid substance.^8^ Adsorption is a practical technique for removing pollutants from aqueous media due to its low cost, ease of operation, flexibility, and absence of byproduct formation.^9^ However, adsorption has limitations, such as variability in the performance of certain inorganic adsorbents, challenges in solid–liquid separation with carbon powder adsorbents, and difficulties in regenerating adsorbents.^10^ Bioremediation is an eco-friendly and cost-effective technique that uses microorganisms to degrade glyphosate through metabolic processes such as oxidation, reduction, and hydrolysis. Despite its potential, bioremediation faces challenges, including reliance on environmental conditions that can inhibit microbial activity, the need for effective microbial strains (which may require the introduction of additional strains), and the persistence of glyphosate in the environment.^11^ Additionally, the degradation process may produce toxic byproducts, and scaling up from laboratory studies to field applications can pose logistical difficulties.^12,13^ Among chemical techniques, electrochemical and photoelectrochemical (PEC) degradation are emerging as sustainable and effective approaches.^14–16^ These methods use redox reactions and solar energy, along with externally applied potentials, to drive nonspontaneous degradation of pollutants (positive Gibbs free energy, Δ*G* > 0).^2^ Another promising strategy involves converting glyphosate at zero bias, effectively generating power in a fuel cell.^17^ This is achievable when the Gibbs free energy is negative (Δ*G* < 0), which corresponds to a positive cell voltage (*E*_cell_ > 0).^18^ This condition is met when the reduction reaction occurs at a potential more positive than that of the oxidation reaction, ensuring an energetically favorable process.

A promising and unexplored technology to convert energy from glyphosate is the microfluidic fuel cell (μFC). The μFCs generate electricity by allowing fuel and oxidant to flow parallel to the electrodes, without mixing, eliminating the need for a physical membrane to separate these liquids.^19–22^ This design takes advantage of microchannels, which operate at low Reynolds numbers (Re < 100), effectively addressing the mass transport issues present in conventional fuel cells.^21,23–25^ Flow-through architectures, mixed-media configurations, and adaptable reactant environments are just a few of the benefits that μFCs provide,^20,24,26^ which increase their efficiency and versatility. But to further enhance their performance, more active anode materials are still needed.

Using advanced materials, Gomes et al. proposed a direct glyphosate photo fuel cell with a dual-modified hematite photoanode to generate electricity,^17^ showcasing the mean of degrading glyphosate at zero bias while generating power. Although the authors demonstrated the feasibility of converting energy from glyphosate with degradation and mineralization of the pollutant at zero bias, there remains a critical need for developing new anodes and reactor architectures to optimize the process and increase efficiency.

The use of metallic catalysts for glyphosate electrooxidation remains largely unexplored, primarily due to the high overpotentials required to drive the reaction, which hinders the development of practical fuel cells. However, by employing a μFC, it is possible to optimize each half-cell reaction under different pH conditions, thereby opening new opportunities for mixed-media energy conversion systems.^25,27,28^ While this approach has not yet been extensively explored in electrocatalysis, Ni-based anodes have been successfully applied in the analytical detection of glyphosate.^29,30^ In 2008, for the first time, Sierra et al. reported glyphosate electrooxidation on Ni in an alkaline medium.^29^ Most recently, Gonçalves et al. used crumpled graphene modified with Ni nanoparticles for glyphosate detection.^30^ Catalysts with low metal loading and high dispersion show better turnover frequencies and cost-efficiency than bulk forms.^31^ Studies on sputtered Ni nanoclusters for various electrocatalytic reactions^32,33^ suggest that this technique can enhance efficiency by preparing well-dispersed Ni catalysts.

This study explored using a Ni anode with minimal content and a metal-free cathode to convert glyphosate into energy. In this sense, we present a novel μFC design featuring a nickel-supported carbon paper (Ni/CP) anode and a metal-free carbon paper (CP) cathode, which is aimed at generating electricity while mitigating glyphosate. To reduce the amount of nickel used and minimize the overall cost of the device, we employ magnetron sputtering to directly deposit Ni onto the CP surface.^34,35^ This approach produces CP-supported Ni nanoclusters capable of electrooxidizing glyphosate in an alkaline medium. The anodic reaction is paired with HClO reduction on a bare CP cathode to generate power. This innovative strategy demonstrates an efficient method for converting energy from glyphosate, a widely used agricultural herbicide.

## 2 Experimental Section

### 2.1 Synthesis and Characterization of the Ni/CP Electrodes

For electrodes preparation, we used the magnetron sputtering coating apparatus which consists of several key components, including a sputtering deposition system, DC power supply, gas inlet system, vacuum system, cooling system, and control system.^36^ Figure 1A provides an illustrative depiction of the electrode preparation process using sputtering. The experiment utilized a single circular plane Ni target, 50.8 mm diameter, and a maximum thickness of 1.5 mm, with a purity of 99.995% procured from Zhengzhou CY Scientific Instrument Co., Ltd. Ni nanoclusters were deposited onto flame-annealed-treated CP, with high-purity argon as the plasma-forming gas. The base pressure was ∼5 × 10^–5^ Pa, and the Ni target was sputtered using a DC power supply set to 50 W, a gas flow rate of 50 SCCM, and deposition times of 20, 40, 60, and 80 s for each side of CP. CP samples were positioned perpendicularly and centered 7.5 cm from the target. No rotation was applied, and the CPs were sputtered in the stationary mode. Before Ni/CP synthesis, the target was presputtered for 1 min at the same power to remove any surface residue.

**Figure 1:**
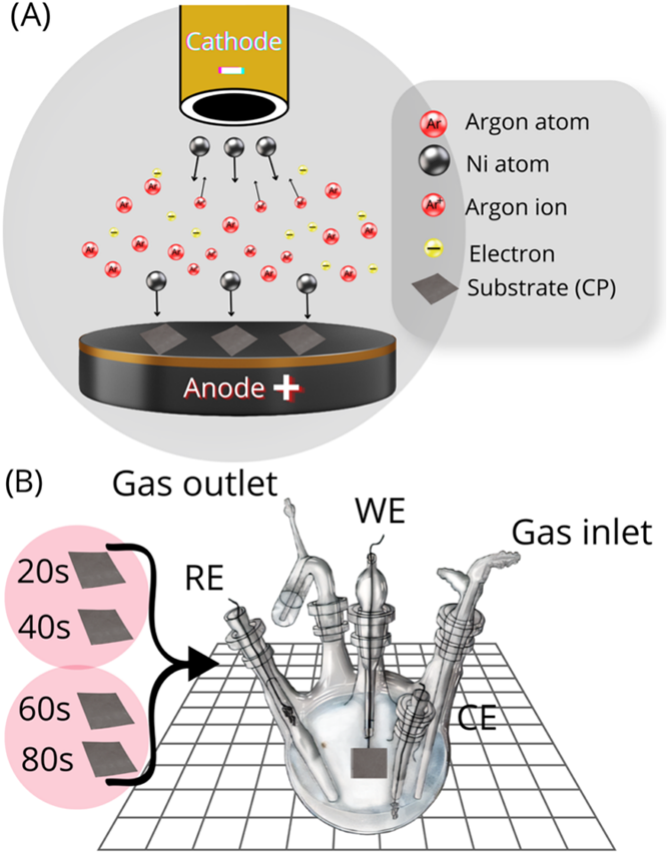
(A) Illustration of electrode preparation using magnetron sputtering and (B) the configuration of a three-electrode electrochemical cell used to investigate the electroactivity of Ni/CP toward glyphosate electrooxidation.

The samples were labeled according to the specific deposition time, with ″Ni/CP_*x*″ indicating that the nickel target was sputtered for *x* seconds under the specified conditions.

The amount of Ni deposited on CP was qualitatively investigated using an X-ray spectrometer EDS from Oxford Instruments, model Xplore 15 mm^2^, coupled to a scanning electron microscopy SEM, JEOL model JSM6380LV. The Ni nanoclusters were identified and studied using a double-corrected transmission electron microscope (dc-TEM) from Thermo Fisher/FEI, model Titan Cubed Themis, at the Brazilian Nanotechnology National Laboratory (LNNano). The Ni/CP electrode was ground in a mortar until a fine, homogeneous powder was obtained. The powder was dispersed in 2-propanol and deposited on the carbon-coated Cu grid before introduction in the dc-TEM.

### 2.2 Electrochemical Measurements

The Ni/CP_*x* electrodes were precisely cut to 3 × 9 mm, operating with a portion of 3 × 6 mm (0.18 cm^2^) of immersed geometric area for half-cell measurements and used for electrochemical current normalization. A 5% Nafion solution was applied to the nonimmersed (3 × 3 mm) upper contact area of the Ni/CP_*x* to make it hydrophobic, thereby avoiding electrolytes soaking and reaching the toothless crocodile contact. We used a conventional three-electrode configuration cell for half-cell measurements, as illustrated in Figure 1B. The Ni/CP_*x* electrode served as the working electrode (WE), while the platinum and Ag/AgCl electrodes functioned as the counter electrode (CE) and reference electrode (RE), respectively.

We used two different commercial glyphosate products from Insetimax: one with a lower concentration (1%) and another with a higher concentration (45%). Glyphosate residues in water are typically found at low concentrations, which creates challenges in their degradation using physical or biological methods. To address this, we selected concentrations similar to those commonly found in various water sources to ensure their practical relevance. We prepared two glyphosate solutions: one more diluted with a concentration of 16.2 ppm (pH 13.70), and another more concentrated with 29.6 ppm (pH = 14.25). The 16.2 ppm solution was prepared from the less concentrated (1%), and the 29.6 ppm solution was prepared from the concentrated one (45%). The concentrations were measured using high-performance liquid chromatography (HPLC), ensuring precise values. These concentrations surpass the World Health Organization’s recommended limit for acceptable glyphosate levels in groundwater (0.9 mg L^–1^).^37^ This allowed us to investigate the degradation process under conditions of elevated contamination, simulating scenarios where glyphosate levels exceed safe thresholds.

The half-cell experiments were performed in an O_2_-free 1.0 mol L^–1^ KOH in the absence and presence of the two glyphosate solutions. The Ni/CP anodes 0.18 cm^2^ immersed in electrolyte were used as working electrodes, a Pt plate as counter electrode, and Ag/AgCl as a reference. The potential window was maintained between 0.1 and 0.6 V vs. Ag/AgCl to avoid the oxygen reduction reaction (OER). The conventional three-electrode setup was interfaced with a 5000E Gamry Instruments Potentiostat/Galvanostat Interface 5000E Gamry Instruments. Measurements were performed at a scan rate of 0.02 V s^–1^ and under controlled room temperature conditions (25 ± 1 °C). Deionized water obtained from a Millipore Milli-Q system was used throughout the experiments to ensure a high purity.

### 2.3 Additive Manufacturing of the Microfluidic Fuel Cell

The μFC design, originally developed by our group, has been validated as a proof of concept in a flow-through microfluidic fuel cell configuration.^38^ The fabrication approach involves 3D printing a μFC model specifically designed to house the porous electrodes, positioning the electrodes in place, and sealing the flat areas with an adhesive layer sourced from a touchscreen protector and a glass cover. Briefly, the μFC configuration is constructed using 190-μm-thick carbon paper (Toray Paper 060) integrated into a 3D-printed μFC with a microchannel depth of 150 μm. The adhesive layer, which is attached to the 3D-printed cell, is exposed, allowing for the attachment of Cu wires to the upper section of the device. Porous electrodes are then carefully placed into their designated grooves for the cathode and anode. Subsequently, the outer plastic film from the touchscreen protective piece is removed to reveal a highly adhesive surface, enabling the glass layer to be attached securely and effectively sealing the μFC. To ensure proper connectivity, the Cu wires and porous electrodes are bonded using Ag epoxy, which is left to dry naturally for 24 h vacuum drying process (Figure S1), completing the μFC assembly. The plastic front part of the cell was modeled using Autodesk Inventor 2023 software, printed in a Stereolithography Apparatus (SLA) photosensitive resin type, and 3D cured in 404 nm in an Anycubic model Photo Mono X. Figure 2 illustrates the steps of μFC assembling. More details are provided elsewhere.^38^

**Figure 2:**
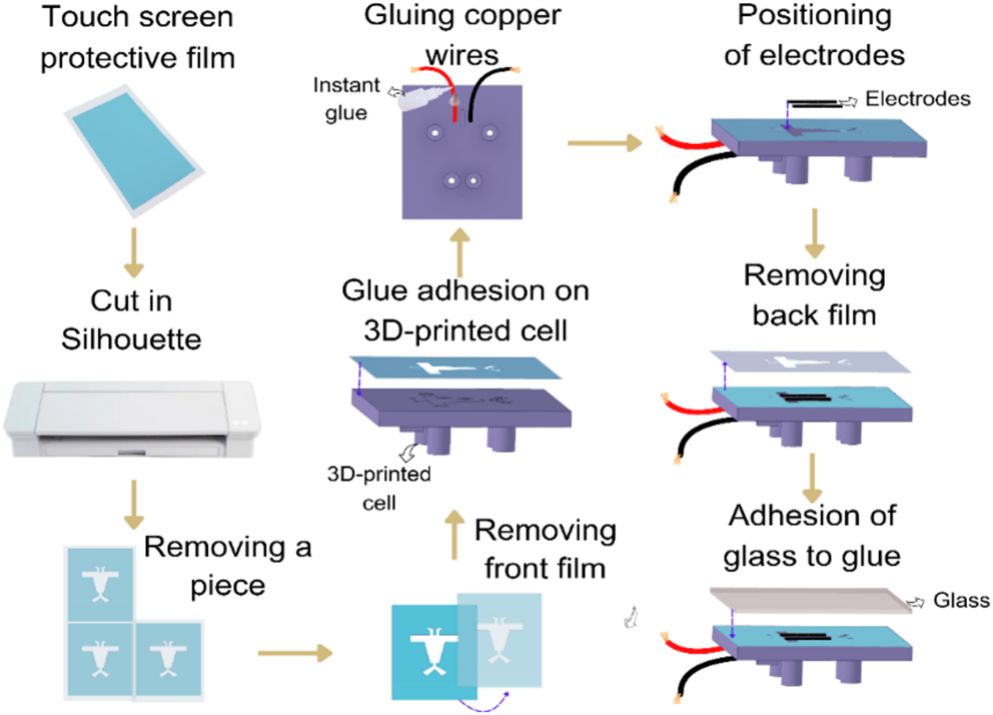
Illustrative steps for assembling the μFC, including cutting the touchscreen protective film, positioning the electrodes, and securely sealing the device.

### 2.4 Microfluidic Fuel Cell Tests

The μFC tests were performed with Ni/CP anode supplied with an anolyte of 16.2 (29.6) ppm glyphosate +1.0 mol L^–1^ KOH, while a metal-free CP cathode was fed with 1.0 mol L^–1^ H_2_SO_4_ in commercial NaClO (HClO) as the catholyte, a strong oxidant. The performance of these mixed-media systems was evaluated in terms of polarization and power density curves, using liquids driven by a dual syringe pump (KDS Legato, model 101) at flow rates of 10, 50, and 100 μL min^–1^. A Potentiostat/Galvanostat Interface 5000E Gamry Instruments was used for all cell measurements, recording the current response from open-circuit voltage (OCV) to 0.001 V at a scan rate of 0.01 V s^–1^. Figure S2 provides a detailed view of the fully assembled apparatus used for the cell measurements. Under the same configuration, the μFC was evaluated for long-term stability (3 h) under potentiostatic conditions. The anode cross-sectional area of 0.015 cm^2^ was used to normalize current and power.

The glyphosate conversion in potentiostatic experiments was investigated by HPLC conducted using a Shimadzu HPLC system (SPD-M20A) equipped with a Zorbax ODS Agilent column (5 μm, 4.6 × 150 mm) operating at a wavelength of 260 nm at 35 °C. We used methanol:water (70:30 v/v) pH 3 as an eluent at 0.8 mL min^–1^. All samples were filtered and placed in appropriate tubes for the HPLC analysis. Following the stabilization of experimental conditions (temperature and pressure), measurements were performed, with a focus on integrating the peak around 230 nm. The resulting data were compared to the calibration curve to ascertain the exact concentrations of the samples.

## 3 Results and Discussion

### 3.1 Electrode Characterization

The high-angle annular dark-field scanning transmission electron microscopy (HAADF-STEM) images shown in Figure 3 illustrate the deposition of Ni on CP using sputtering for two different exposure times, 20 s (Ni/CP_20, Figure 3A–C) and 60 s (Ni/CP_60, Figure 3D–F). The images show cluster formations of a few nanometers and clearly reveal atomic-level deposition and some well-defined Ni nanoclusters (Figure 3B,D) formed by atoms, as shown in the inset of Figure 3B. Figure 3C,D,E shows Ni atoms (white dots in the image) deposited on the surface of carbon paper structures. The nanoclusters built from Ni atoms form naked particles,^39^ with no surface contaminants,^40,41^ usually found by chemical synthesis.^42,43^ This cleanliness is a hallmark of sputtering techniques, which are known for producing high-purity surfaces without requiring additional treatments.^44^ Such clean and active sites are critical for electrochemical applications where surface contamination can significantly reduce performance.

**Figure 3:**
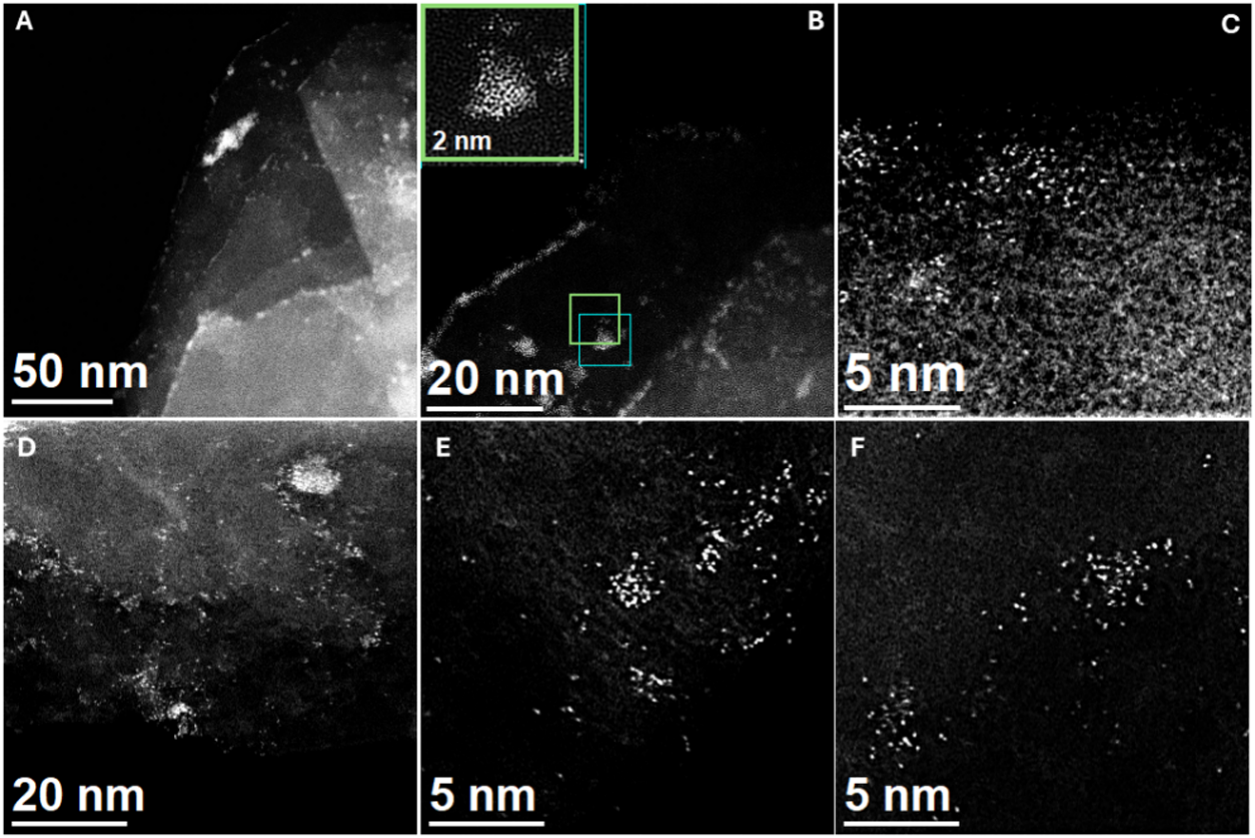
High-angle annular dark-field scanning transmission electron microscopy (HAADF-STEM) images of (A–C) Ni/CP_20 and (D–F) Ni/CP_60. The inset in image 3B is a zoomed-in view of a nanocluster forming of Ni atoms.

The comparison between Ni/CP_20 and Ni/CP_60 suggests that the longer sputtering time (60 s) results in either more dispersed or slightly larger clusters compared to the shorter sputtering time (20 s). This observation aligns with established principles of sputtering deposition, where prolonged exposure typically increases the amount of material deposited, potentially influencing the cluster size and distribution. Importantly, the nanoclusters appear to be well-separated with minimal agglomeration, demonstrating control over the deposition process. This characteristic is advantageous for applications requiring high surface activity as the increased active surface area promotes more efficient catalytic reactions.

It is worth noting that despite the success in visualizing these nanostructures, the low amount of nickel deposited presents a challenge for further characterization. Many conventional techniques, such as X-ray diffraction, X-ray photoelectron spectroscopy, and Raman spectroscopy, may lack the sensitivity needed to detect such small amounts of material. Therefore, we tentatively explored EDS spectra as an alternative way to investigate the materials.

Semiquantitative analysis of the average EDS spectra obtained from the 4 electrodes presented loadings (wt %) of 0.14, 0.31, 0.46, and 0.58 for Ni/CP_20, Ni/CP_40, Ni/CP_60, and Ni/CP_80, respectively. Details are provided in Figure 4. The EDS spectra reveal distinct peaks corresponding to nickel in the expected energy range (∼7.5 keV for the Ni Kα line), confirming the successful deposition of Ni on the carbon substrate. The comparison across samples shows slight variations in the intensity of the Ni peaks, as highlighted in the zoomed-in inset (Figure 4). This suggests differences in the amount of nickel deposited, which are likely influenced by varying sputtering times or conditions.^41,45^ Samples with longer sputtering times typically exhibit stronger Ni peaks, correlated with a higher amount of deposited material. This trend supports the controlled deposition achieved through sputtering, in which the process parameters directly influence the outcome.

**Figure 4:**
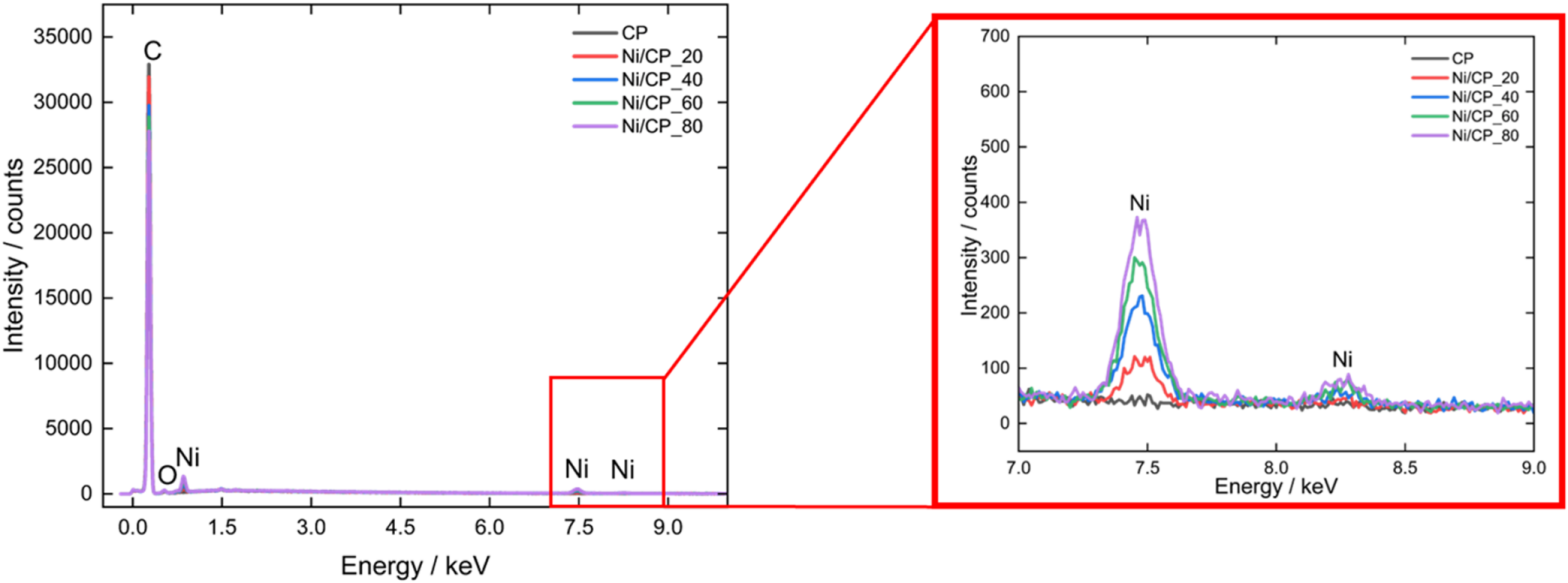
EDS spectra and qualitative analysis of the Ni/CP_*x* electrodes.

The spectra also highlight a dominant carbon peak, which is expected, given the CP substrate and the relatively low Ni loading. Despite this, the Ni peaks remain clearly distinguishable, even with a modest signal-to-noise ratio. Furthermore, the spectra do not show any significant peaks for elements other than Ni and C, indicating that the sputtering process did not introduce notable impurities. This result highlights the cleanliness and precision of the deposition method.

While EDS is not inherently quantitative at low levels, the relative intensities of the Ni peaks corroborate the TEM findings, confirming the successful formation of Ni nanoclusters on the CP. The consistency of these results across both techniques further validates the reliability of the sputtering process.

### 3.2 Half-Cell Measurements

Prior to energy conversion investigation, we studied the electrocatalytic parameters of Ni/CP toward glyphosate electrooxidation in an alkaline medium. Figure 5A displays the electrochemical profile of Ni/CP_*x* in 1.0 mol L^–1^ KOH. The cyclic voltammograms show distinct redox peaks for the four electrodes. These peaks correspond to the typical electrochemical activity of nickel-based electrodes in alkaline environments, including the oxidation of Ni­(II) to Ni­(III) (Ni­(OH)_2_ to NiOOH) and subsequent reduction.^46^ The double layer region of nickel ranges from 0.1 to 0.35 V vs. Ag/AgCl. The range between 0.35 and 0.48 V vs. Ag/AgCl, where the electrode surface is primarily covered by Ni hydroxide, as described by eq 1 ^47,48^

1 $$\mathrm{Ni}+2{\mathrm{OH}}^{-}↔\mathrm{Ni}{\left(\mathrm{OH}\right)}_{2}+2e^{-}$$

The region from 0.48 to 0.6 V vs. Ag/AgCl involves the Ni­(II)/Ni­(III) redox couple, described by eq 2 ^49,50^

$$\mathrm{Ni}{\left(\mathrm{OH}\right)}_{2}+{\mathrm{OH}}^{-}↔\mathrm{NiOOH}+H_{2}O+e^{-}$$ 2

In the backward scan, NiOOH is reduced back to Ni­(OH)_2_. This redox cycling between NiOOH and Ni­(OH)_2_ is the foundation of Ni’s catalytic activity in alkaline environments, especially for pollutant degradation.^29^ The variation in current responses across the electrodes may reflect differences in their electroactive surface areas, Ni loading, or surface modifications. Electrodes exhibiting higher current densities are indicative of greater catalytic activity. At first sight, it seems like the Ni/CP_60 shows the highest surface area (Figure 5A), which is transduced in activity (Figure 5B). In contrast, Ni/C_80 profile would lead us to rationalize that there is less surface area (Figure 5A); however, it shows high activity toward the anodic reaction (Figure 5B). These results highlight the complexity of investigating nanoclusters deposited at an atomic level on highly porous carbon structures. Therefore, the electrochemical profiles are rather used to identify the Ni on CP than to draw a fine conclusion about surface condition.

**Figure 5:**
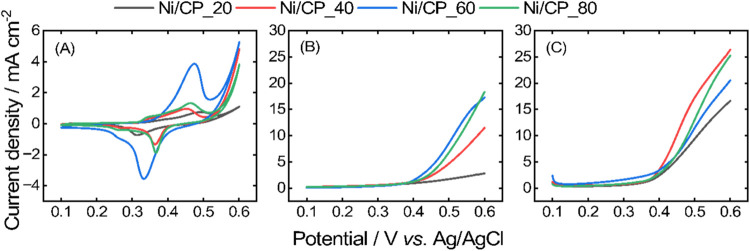
Cyclic voltammograms for Ni/CP electrodes in 1.0 mol L^–1^ KOH (a) in the absence of glyphosate; (b) in the presence of 16.2 ppm; and (c) in the presence of 29.6 ppm glyphosate. Experiments were performed at a scan rate of 0.02 V s^–1^.

The positive potential going scan part of the voltammograms obtained in the presence of 16.2 ppm (Figure 5B) and 29.6 ppm (Figure 5C) glyphosate in 1.0 mol L^–1^ KOH depict the increase in current density compared to the absence of the organic (Figure 5A). This result proves the electrooxidation of glyphosate catalyzed by the active Ni nanoclusters. The oxidation is facilitated by the formation of Ni­(III) species, which are highly reactive in alkaline conditions and are known to break down organophosphate bonds.^29^ As shown in Figure 5A, the transition from Ni­(II) to Ni­(III) occurs at approximately 0.5 V (vs. Ag/AgCl), which corresponds to the potential where the Ni/CP_*x* electrodes begin to exhibit significant catalytic activity, as demonstrated in Figure 5B,C. These figures further illustrate the effect of the sputtering deposition time on the performance of the Ni/CP_*x* electrodes. From Figure 5B at 0.5 V, as deposition time increases from 20 to 60 s, the current density rises gradually from 1.92 to 8.80 mA cm^–2^, attributed to the increased availability of active sites. However, beyond the 60 s, extending the deposition time to 80 s leads to a 21.9% reduction in current density (6.87 mA cm^–2^) due to possible Ni atoms aggregation, as suggested in the previous studies.^41,45^ The electrochemically active surface area (ECSA), calculated by integrating the Ni oxides reduction peak (at around 0.45–0.20 V) using 257 μC cm^–2^ as the specific charge,^51,52^ is presented in Table S1. The ECSA increases from 2.11 to 11.3 cm^2^ with sputtering times ranging from 20 to 60 s, finally decreasing to 4.07 cm^2^ with 80 s. This pattern reflects the electroactivity.

The voltammograms with a higher glyphosate concentration (29.6 ppm) further confirm the concentration-dependent nature of the anodic reaction (Figure 5C). The increased current densities at higher glyphosate concentrations indicate enhanced reactivity at the electrode surfaces, as more reactant is available to participate in the oxidation reaction.^24^ Figure S3 shows the current densities at 0.5 V for all electrodes in both glyphosate concentrations. For instance, for the Ni/CP_60 electrode, the current density rises from 8.80 to 10.39 mA cm^–2^ with an increase in concentration. The highest current density appears for Ni/CP_60 in the diluted glyphosate solution, but for Ni/CP_40 in the concentrated one. This suggests that at higher glyphosate concentrations, surface poisoning or oversaturation occurs, where excessive Ni loading (as in Ni/CP_60) may exacerbate active site blockage. In contrast, at lower concentrations, the reduced fouling allows Ni/CP_60 to utilize its higher ECSA effectively. This indicates that 40–60 s sputtering provides optimal Ni coverage depending on fuel concentration, and that current output scales with glyphosate availability despite surface effects.

The onset potential of the anodic reaction (*E*_onset_^A^) can be estimated by the derivative voltammogram.^53,54^ At 16.2 ppm, the onset is displaced toward lower values while the increase from 20 to 60 s and increases again for Ni/CP_80 (Figure 5B). At a 29.6 ppm concentration of glyphosate, *E*_onset_^A^ is virtually the same for all electrodes (Figure 5C). Ni/C_60 shows the highest current density and the lowest onset of 0.36 V for the diluted solution (Figure 5B), as shown in Figure S4, ascribed to its high ECSA and active sites availability. Therefore, this electrode is used to equip the glyphosate μFC.

To achieve a galvanic cell, this anodic reaction must be coupled to a cathodic reaction with a higher onset potential (*E*_onset_^C^). This is achieved by employing a metal-free CP cathode for the reduction of HClO in 1.0 mol L^–1^ H_2_SO_4_ as the cathodic half-cell reaction, as previously reported by Guima et al.,^25^ since *E*_onset_^C^ is 0.99 V vs. Ag/AgCl.^25^ A liquid oxidant like HClO addresses solubility issues that gaseous oxidants face, enhancing utilization and preventing oxidant starvation in fuel cells. Additionally, HClO can be produced by acidifying commercial bleach solution.^55^ Under these conditions, a μFC is constructed with a theoretical potential of *E*_cell_ = 0.63 V (*E*_cell_ = *E*_onset_^C^ – *E*_onset_^A^). Considering that Δ*G* = −*nFE*_cell_, we find Δ*G* < 0, a spontaneous coupled reaction in a galvanic μFC. Where n is the number of mols of electrons and F is the Faraday’s constant.

### 3.3 Microfluidic Fuel Cell Tests

After exploring various sputtering times for preparing CP modified with nickel nanoclusters and identifying the most active catalyst for glyphosate electrooxidation suitable for constructing a galvanic cell, we proceeded to conduct cell performance tests. Figure 6A,C shows the polarization and power density curves for the mixed-media glyphosate/HClO μFC with a Ni/CP_60 anode and CP cathode in flow-through configuration. The OCV is ∼0.5 V when fed by 16.2 ppm and ca. 0.3–0.4 V for 29.6 ppm. They are both lower than the 0.63 V theoretically expected. This decrease is a consequence of unavoidable practical conditions, involving connection and fluid mechanics. Among the two glyphosate concentrations, the diluted solutions seem to lead toward higher OCV, but the limited fuel produces less power density.

**Figure 6:**
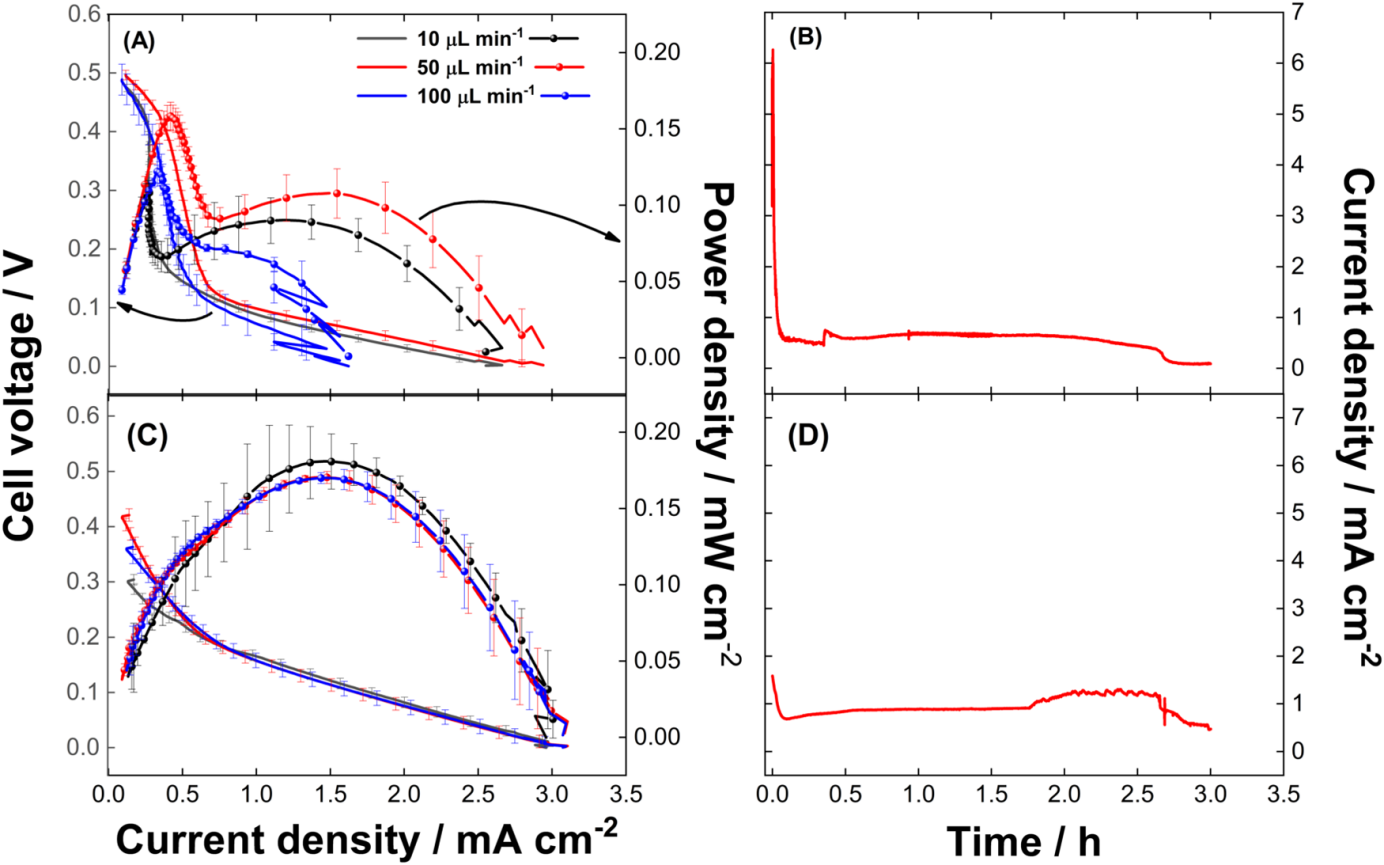
Polarization and power density curves at different flow rates for the microfluidic fuel cells fed by (a) 16.2 ppm of glyphosate in 1.0 mol L^–1^ KOH anolyte and (b) their respective chronoamperometry at 0.45 V. Also features the cell fed by (c) 29.6 ppm of glyphosate in 1.0 mol L^–1^ KOH and (d) their respective chronoamperometry at 0.185 V. All cells are fed by 1.0 mol L^–1^ H_2_SO_4_ in HClO as catholyte, equipped with Ni/CP_60 anode and CP cathode at 50 μL min^–1^.

The polarization curves (left-hand *Y*-axis in Figure 6A,C) are predominantly influenced by ohmic polarization with notable differences observed between cells fed with varying glyphosate concentrations. This behavior aligns with findings by Kjeang et al., who demonstrated that ohmic losses significantly impact flow-through configurations.^21^ At a glyphosate concentration of 16.2 ppm (Figure 6A), activation polarization is less pronounced, as indicated by a more stable cell voltage at low current densities. In contrast, at 29.6 ppm (Figure 6C), the voltage drops sharply in the low-current region, reflecting stronger activation losses. This behavior is likely associated with the saturation of electroactive sites or partial poisoning by glyphosate or its intermediate oxidation products. Based on established mechanisms reported for small organic molecule electrooxidation,^24,56^ we hypothesize that at higher glyphosate concentrations, excessive surface coverage by the reactant or its partially oxidized intermediates hinders the availability of active Ni­(III) sites required for effective dissociative adsorption and oxidation. Conversely, in more diluted anolytes, surface renewal is favored, allowing for easier desorption of byproducts and facilitating continuous catalytic turnover.^57^ Figure 6A,C shows the expected behavior for polarization curves, characterized by a monotonic decrease in cell voltage with increasing current density. Additionally, the absence of a limiting current density suggests that there is sufficient HClO oxidant available to accept electrons from the anode. This behavior is typical for organic fuels, implying that the oxidation process is not entirely efficient with low Ni loading on the Ni/CP electrodes.

Figure 6C shows that OCV varies with flow rate, suggesting that the colaminar interface is more stable at 50 μL min^–1^, while the low flow rate of 10 μL min^–1^ may lead to reduced interface stability. Besides the OCV, the polarization curves at 29.6 ppm glyphosate are not significantly affected by flow rate, indicating that overall energy conversion remains governed by fuel availability rather than interface dynamics under load. However, at 16.2 ppm glyphosate (Figure 6A), the flow rate significantly impacts performance. Increased mass transport at flow rates between 10 and 50 μL min^–1^ enhances the cell performance but decreases at 100 μL min^–1^. This decline is attributed to the competition between improved mass transport and reduced residence time. In the concentrated anolyte, the decrease in residence time at 100 μL min^–1^ does not negatively impact cell performance, as the greater availability of reactants ensures a sufficient supply to the electrode. Even with the increased flow rate, enough reactants still reach the electrode to sustain electrochemical reactions effectively.

The inverse relationship between flow rate and current density for the μFC fed by 16.2 ppm glyphosate (2.94 mA cm^–2^ at 50 μL min^–1^ vs. 1.62 mA cm^–2^ at 100 μL min^–1^) highlights residence time limitations at low concentrations. Conversely, with the μFC fed by 29.6 ppm glyphosate, sustained higher current density of ca. 3.07–3.08 mA cm^–2^ across 50–100 μL min^–1^. This is explained by the higher reactants availability which compensates the diffusion constraints.^58^ In this case, the performance is less influenced by variations in flow rates, because reactant availability is no longer the primary limiting factor. Instead, other factors such as polarization losses and internal resistance become more significant, as discussed previously.

The power density curves (right-hand *Y*-axis in Figure 6A,C) further highlight the performance characteristics of the cells. At 16.2 ppm glyphosate as the anolyte, the cell voltage at the maximum power density (*P*_max_) reaches 0.40, 0.38, and 0.37 V for 10, 50, and 100 μL min^–1^, respectively. The maximum power density reaches 0.11 mW cm^–2^ at 10 μL min^–1^ and 0.12 mW cm^–2^ at 100 μL min^–1^ flow rates but increases to 0.16 mW cm^–2^ at 50 μL min^–1^. In contrast, the behavior changes significantly at 29.6 ppm glyphosate (Figure 6C), where *P*_max_ is 0.18 mW cm^–2^ at 0.13 V for 10 μL min^–1^ and 0.17 mW cm^–2^ at 0.12 V for both flow rates of 50 and 100 μL min^–1^. It is worth noticing that a dual-peak profile observed in the polarization curve (Figure 6A) likely results from the interplay between kinetic and mass transport effects in the μFC, a behavior previously reported in similar μFC systems fed by glycerol and CO_2_.^38^ The first and main peaks represent the controllable part of the operating system and indicate the highest power density that can be reached.

At lower glyphosate concentration, a flow rate of 50 μL min^–1^ results in a higher power density and an increased OCV, effectively minimizing diffusion limitations and enhancing overall performance. In contrast, at higher glyphosate concentrations, the cell voltage remains largely unaffected by the flow rate, as previously discussed. However, while the highest power density (0.18 mW cm^–2^) is observed at 10 μL min^–1^, a flow rate of 50 μL min^–1^ provides a slightly lower power density, making it a more balanced operating condition depending on the application. This nuanced relationship among flow rate, power density, and cell voltage emphasizes the importance of optimizing flow conditions for efficient cell operation.

This study represents the first time that an μFC has been powered by glyphosate. The cell parameters are detailed in Table S2, along with a comparison to similar systems utilizing metal-free CP cathodes but equipped with anodes containing high amounts of noble or non-noble metals. The findings demonstrate that even with low nickel loading on the anode, the system performs competitively, offering a novel approach to μFC design and glyphosate electrooxidation.

Previous studies have primarily focused on combining advanced oxidation processes with biodegradation in microbial fuel cells. High degradation efficiencies superior to 70% have been achieved through electrochemical and bioelectrochemical approaches in both single- and two-compartment fuel cells.^17,57,59,60^ Carrera-Cevallos et al. reported electrochemical degradation of glyphosate up to 99% in a single-compartment cell equipped with boron-doped diamond electrodes, though this method was limited by its high environmental footprint and economic cost.^61^ Meanwhile, the FePc-rGO/ACF electrode, incorporating iron phthalocyanine (FePc) and reduced graphene oxide (rGO) dispersed in activated carbon fiber (ACF), exhibited excellent electrochemical performance in a microbial fuel cell (MFC), achieving a maximum current density of 8050 mA m^–2^ and a power density of 1101 mW m^–2^, while enabling ∼80% glyphosate degradation via an efficient four-electron oxygen reduction reaction at the cathode.^60^ In this two-compartment MFC, a bacterial culture spiked with 30 mg L^–1^ of glyphosate in the anode chamber facilitated pollutant degradation. Furthermore, Gomes et al. recently employed modified hematite in a glyphosate-fueled photocatalytic fuel cell, a two-compartment system that achieved both energy generation and 98% glyphosate degradation via photoelectrochemical processes.^17^ To the best of our knowledge, this study is the first to utilize glyphosate as a direct fuel, leveraging the advantages of a microfluidic fuel cell over conventional batch systems while achieving glyphosate degradation at zero bias.

Since 50 μL min^–1^ seems to properly balance mass transport and residence time, we conducted chronoamperometry to assess the stability of the μFC over 3 h. For both scenarios, the working power density was set to 4/5 *P*_max_, and the optimal working potentials were determined to be 0.45 and 0.185 V for the less concentrated and more concentrated glyphosate solutions, respectively. As shown in Figure 6B,D, a current drop was observed during the initial minutes for both concentrations. The μFC fed with the more concentrated glyphosate demonstrated greater stability and a higher current density (Figure 6D), averaging 0.9 mA cm^–2^, compared to 0.6 mA cm^–2^ for the less concentrated glyphosate solution (Figure 6B). The higher current density in the more concentrated solution can be attributed to the increased availability of reactants. This trend in the current density aligns with the potentiodynamic results discussed earlier. These findings highlight the stability and practical potential of the μFC equipped with Ni/CP as the anode and CP as the cathode for glyphosate degradation coupled with energy conversion.

### 3.4 Glyphosate Degradation

The electrochemical conversion of glyphosate into energy was investigated by analyzing the outlet of the μFC after 180 min of chronoamperometry using the two glyphosate concentrations. Samples were injected into the HPLC instrument for analysis. The relationship between concentration and peak area was established using an external calibration curve, covering a range of 0.05 to 25 mg L^–1^. The calibration curve exhibited excellent linearity with a regression coefficient (*R* ^2^) of 0.9949.

Rather than analyzing the separate outlets from the μFC, samples were collected by mixing the anolyte and catholyte before (denoted as Gly,B) and after (denoted as Gly,A) passing through the μFC. The initial glyphosate concentrations prepared and confirmed via HPLC were 16.2 ppm for the diluted solution and 29.6 ppm for the concentrated solution, achieved by mixing the anolyte and catholyte in a 1:1 ratio. This approach allows for a direct comparison with the mixed outlet solution after passing through the μFC, enabling an accurate assessment of the glyphosate conversion and energy generation efficiency. The degradation of glyphosate (Gly%) was calculated according to eq 3.

3 $$\mathrm{Gly}\%=\frac{C_{\mathrm{Gly},B}‐C_{\mathrm{Gly},A}}{C_{\mathrm{Gly},B}}\times 100$$

For the diluted anolyte (16.2 ppm), the initial Gly,B concentration of 8.08 mg L^–1^ was converted into nondetectable amounts. Given that the lower limit of quantification is 0.05 mg L^–1^, this indicates a glyphosate conversion efficiency of at least 99.4%. For the concentrated anolyte (29.6 ppm), Gly,B at 14.82 mg L^–1^ was reduced to Gly,A at 2.65 mg L^–1^, corresponding to a conversion efficiency of 82.1%. These findings demonstrate that the μFC, equipped with a low-content Ni/CP anode and a metal-free CP cathode, can achieve an output power of 0.16 mW cm^–2^ with >99% conversion of 16.2 ppm glyphosate, or 0.17 mW cm^–2^ with >82% conversion of 29.6 ppm glyphosate.

While this study focused on investigating glyphosate conversion into energy, it did not directly identify or monitor intermediate byproducts such as aminomethylphosphonic acid (AMPA), which is commonly reported in oxidative degradation pathways. Given the potential environmental persistence and toxicity of AMPA, future work should incorporate targeted analytical techniques such as LC-MS and ion chromatography to identify and quantify possible intermediates and confirm complete mineralization. However, under our operating conditions (moderate potentials, flow-through porous electrode, and strong oxidizing catholyte), we expect further degradation of such intermediates, possibly into inorganic phosphate and other low-molecular-weight species.

Future research should focus on quantifying and identifying the byproducts formed during glyphosate degradation to better understand the process and its environmental implications. This is especially critical as some metabolites, such as aminomethylphosphonic acid (AMPA), are reported to be more toxic than glyphosate itself. Such analysis would not only enhance our understanding of the degradation pathways but also ensure the environmental safety of the process.^62^ Notably, this study demonstrates the capability of the μFC to degrade glyphosate at zero bias to concentrations below the WHO-recommended limit of 0.9 mg L^–1^, offering a promising step toward sustainable and efficient water treatment technologies.

## 4 Conclusions

This work presents a 3D microfluidic fuel cell (μFC) designed to simultaneously degrade glyphosate and generate energy. The system incorporates a nickel-sputtered carbon paper (Ni/CP) anode and a metal-free carbon paper cathode, configured in a flow-through design with glyphosate serving as the anolyte in an alkaline medium and hypochlorous acid (HClO) as the catholyte in an acidic medium. The sputtering process facilitated the precise deposition of high-purity nickel nanoclusters, enhancing the surface activity and catalytic performance while maintaining minimal metal usage.

Electrochemical investigations revealed efficient electrooxidation of glyphosate at varying concentrations, with a notable reduction in the onset potential and an increase in the current density at higher concentrations (29.6 ppm) due to increased reactant availability. Among the tested configurations, the Ni/CP prepared with 60 s achieved a superior catalytic performance, optimizing the balance between active site availability and nanocluster dispersion. This Ni/CP displays 0.36 V vs. Ag/AgCl of onset potential for glyphosate electrooxidation in an alkaline medium. While HClO reduction reaction on bare metal-free CP is 0.99 V vs. Ag/AgCl, we propose coupling these reactions for an OCV of 0.63 V, allowing spontaneous coupled reactions.

The mixed-media μFC equipped with CP/Ni nanoclusters anode and CP cathode achieved glyphosate conversion efficiencies exceeding 99% for diluted solutions (16.2 ppm) and 82% for concentrated solutions (29.6 ppm), reducing glyphosate concentrations to below the World Health Organization’s recommended limit of 0.9 mg L^–1^. Additionally, the μFC delivered a maximum power density of 0.18 mW cm^–2^, effectively coupling pollutant degradation with renewable energy production at zero bias. Chronoamperometric stability tests conducted over 3 h demonstrated the robustness of the system, maintaining high performance.

This innovative glyphosate μFC system addresses significant environmental challenges. Its scalable, cost-effective potential, and sustainable design offer a promising solution for mitigating glyphosate pollution while contributing to renewable energy generation. This work paves the way for future advancements in green technology, environmental remediation, and sustainable energy production.

## Supplementary Material



## Notes

Details of the microfluidic fuel cell; electrocatalytic parameters of glyphosate electrooxidation; cell parameters of the microfluidic fuel cell fed by glyphosate; artificial intelligence through chatbots was utilized to improve grammar and language overall; and Canva AI was used to build the background image of the TOC graphic (PDF)
