# Supplementary material for: Glyphosate Is Converted into Energy in a Microfluidic Fuel Cell Equipped with a Low-Content Ni Anode and a Metal-Free Cathode
Source: ACS Omega. 2025 May 7;10(19):19939–49. doi: 10.1021/acsomega.5c01606 (PMC12096228; doi:10.1021/acsomega.5c01606)
Supplement: Supplementary file 1 [file ao5c01606_si_001.pdf]

Supporting Information

Glyphosate is converted into energy in a microfluidic fuel cell equipped with a low-content Ni anode and a metal-free cathode

Willy Bellard Kira,[a] Daniel F. Costa-Filho,[a] Cinthia R. Zanata,[a] Isabel M. C. de Alcantara,[b] Jefferson Bettini,[c] Flávio L. Souza,[c] Heberton Wender,[a] Cauê A. Martins*[a]

[a]Institute of Physics, Universidade Federal de Mato Grosso do Sul, CP 549, 79070-900, Campo Grande, MS, Brazil.

[b]Institute of Chemistry, Universidade Federal de Mato Grosso do Sul, CP 549, 79070-900, Campo Grande, MS, Brazil.

[c]Brazilian Nanotechnology National Laboratory (LNNano), Brazilian Center forResearch in Energy andMaterials (CNPEM), 13083-100 Campinas, São Paulo, Brazil

*caue.martins@ufms.br

* Corresponding Author. Phone: +55 67 99262 4202

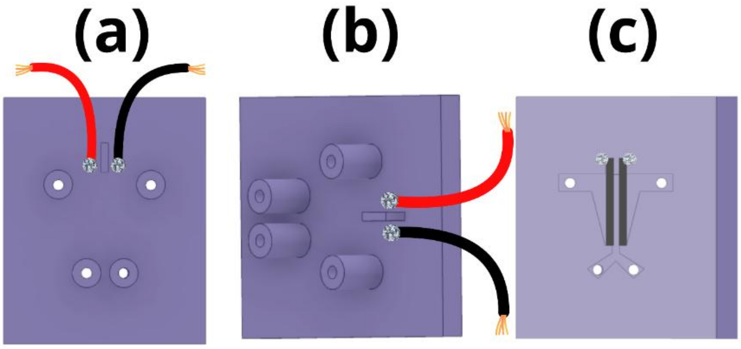

Figure S1. Detailed view of the µFC assembly: (a) backside showing the electrical connections, (b) frontal view highlighting the inlet and outlet, and (c) frontside illustrating the use of silver paste for electrical junctions between the electrodes and electrical wires.

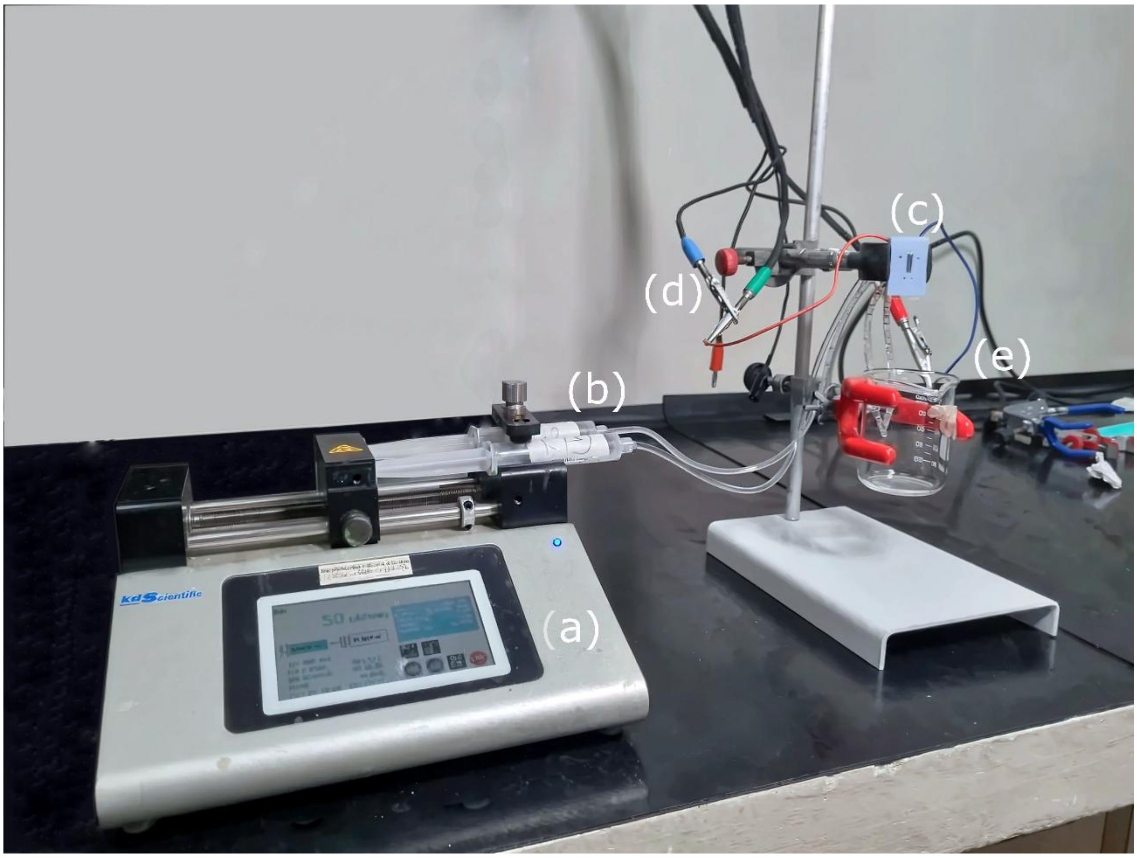

Figure S2. Apparatus setup for µFC measurements: (a) syringe pump, (b) syringes containing the anolyte and catholyte, (c) the µFC, and (d),(e) the connections to the potentiostat.

Table S1. Electrochemically active surface area of Ni/CP_x electrodes, where x is time of exposure to Sputtering.

Electrode	ECSA / cm^2
Ni/CP_20s	2.11E+00
Ni/CP_40s	2.80E+00
Ni/CP_60s	1.13E+01
Ni/CP_80s	4.07E+00

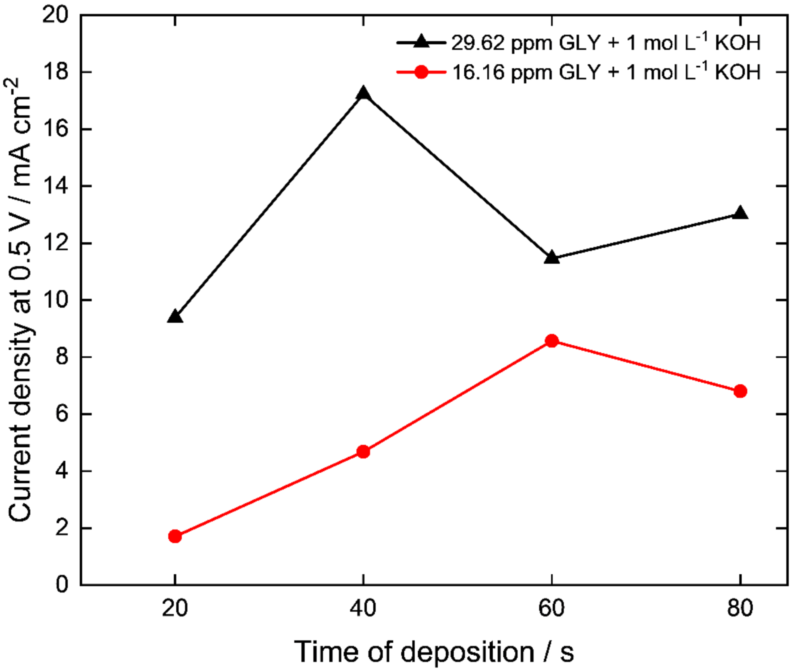

Figure S3. Variation in current density with deposition time at 0.5 V for two different glyphosate concentrations.

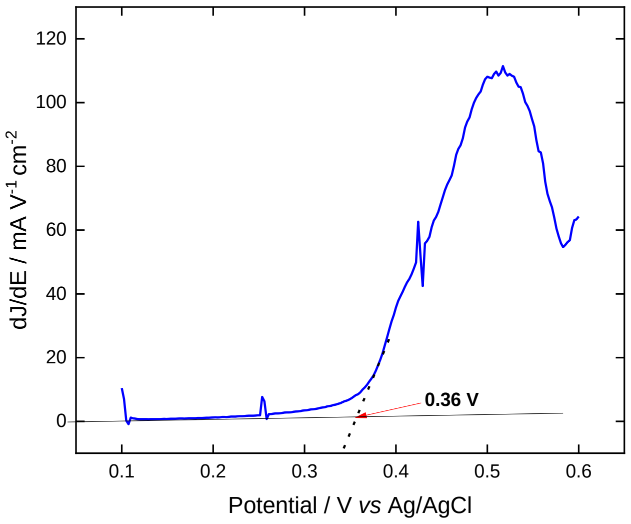

Figure S4. Derivative of voltammogram from the Ni/CP_60 anode for glyphosate oxidation

Table S2. Perfomance parameters of microfluidic fuel cells.

Reference	Flow rate/µL min^{-1}	OCV / V	Maximum power density/ mW cm^{-2}	Cell voltage at maximum power density / V	Anode	Analyte	Cathode	Catholyte
[1]	100	1.2	9.5	0.27	Pt/C/CP	1M Glycerol + 1M KOH	CP	0.5M $Na_2S_2O_8$ + 1M H_2SO_4
[1]	100	1.6	22.5	0.71	Pt/C/CP	1M Glycerol + 1M KOH	CP	Bleach + 1M H_2SO_4
[2]	50	0.25	0.02	0.002358	Cu/CP	1M MeOH + 1M KOH	CP	1M $Na_2S_2O_8$ + 1M H_2SO_4
[2]	100	0.3	0.026	0.003066	Cu/CP	1M MeOH + 1M KOH	CP	1M $Na_2S_2O_8$ + 1M H_2SO_4
[3]	200	2.0	148	0.59	Pd/CP	4M HCOONa + 2M NaOH	CP	1.5M $Na_2S_2O_8$ + 1M H_2SO_4
[4]	200	1.5	89.27	0.64	Pd/CP	0.5M HCOONa + 2M NaOH	CP	0.5M $FeCl_3$+ 1M H_2SO_4
[4]	300	2.0	214.95	0.60	Pd/CP	1.5M HCOONa + 2M NaOH	CP	2M $Na_2S_2O_8$ + 1M H_2SO_4
[5]	200	1.2	8.43	0.18	Ni/CP	1M NH3.H2O + 4M NaOH	CP	1M $Na_2S_2O_8$ + 1M H_2SO_4
This work	10	0.47	0.11	0.40	Ni/CP	16.2 ppm Gly + 1M KOH	CP	HClO+ 1M H_2SO_4
This work	10	0.30	0.18	0.13	Ni/CP	29.6 ppm Gly+1M KOH	CP	HClO+ 1M H_2SO_4
This work	50	0.49	0.16	0.38	Ni/CP	16.2 ppm Gly + 1M KOH	CP	HClO+ 1M H_2SO_4
This work	50	0.42	0.17	0.12	Ni/CP	29.6 ppm Gly +1M KOH	CP	HClO+ 1M H_2SO_4
This work	100	0.48	0.12	0.37	Ni/CP	16.2 ppm Gly + 1M KOH	CP	HClO+ 1M H_2SO_4
This work	100	0.36	0.17	0.12	Ni/CP	29.6 ppm Gly +1M KOH	CP	HClO+ 1M H_2SO_4

References

(1) Guima, K.; Zanata, C. R.; Martins, C. A. Exploring Liquid Oxidants and Metal-free Cathode for Enhanced Performance in a Reusable 3D-printed Glycerol Microfluidic Fuel Cell. *Electroanalysis* **2024**, *36* (2), e202300223. https://doi.org/10.1002/elan.202300223.

(2) Queiroz, B. D.; Vital, P.-L. S.; Budke, K. O.; Rey-Raap, N.; Arenillas, A.; Barra, G. M. O.; Ferreira, D. S.; Camara, G. A.; Wender, H.; Martins, C. A. A Comprehensive Investigation of Methanol Electrooxidation on Copper Anodes: Spectroelectrochemical Insights and Energy Conversion in Microfluidic Fuel Cells. *ACS Appl. Mater. Interfaces* **2024**, *16* (27), 35255–35267. https://doi.org/10.1021/acsami.4c08472.

(3) Lan, Q.; Ye, D.; Zhu, X.; Chen, R.; Liao, Q.; Zhang, T.; Zhou, Y. Direct Formate/Persulfate Microfluidic Fuel Cell with a Catalyst-Free Cathode and High Power Density. *ACS Sustain. Chem. Eng.* **2021**, *9* (16), 5623–5630. https://doi.org/10.1021/acssuschemeng.1c00395.

(4) Liu, C.; Gao, Y.; Liu, L.; Sun, C.; Jiang, P.; Liu, J. High Power Density Direct Formate Microfluidic Fuel Cells with the Different Catalyst-Free Oxidants. *ACS Omega* **2022**, *7* (32), 28646–28657. https://doi.org/10.1021/acsomega.2c03840.

(5) Liu, H.; Xu, Y.; Zhang, S.; Zhou, Y.; Liu, N.; Liu, J.; Lan, Q. High Cost-Performance Direct Ammonia Microfluidic Fuel Cell Benefited from Nickel Nanoparticle Anode and Catalyst-Free Cathode. *Int. J. Hydrog. Energy* **2024**, *69*, 1417–1425. https://doi.org/10.1016/j.ijhydene.2024.05.160.
